# Overexpression of copper/zinc superoxide dismutase from mangrove *Kandelia candel* in tobacco enhances salinity tolerance by the reduction of reactive oxygen species in chloroplast

**DOI:** 10.3389/fpls.2015.00023

**Published:** 2015-01-22

**Authors:** Xiaoshu Jing, Peichen Hou, Yanjun Lu, Shurong Deng, Niya Li, Rui Zhao, Jian Sun, Yang Wang, Yansha Han, Tao Lang, Mingquan Ding, Xin Shen, Shaoliang Chen

**Affiliations:** ^1^Department of Plant Science, College of Biological Sciences and Technology, Beijing Forestry UniversityBeijing, China; ^2^Department of Bio-Instruments, National Engineering Research Center for Information Technology in AgricultureBeijing, China; ^3^Department of Biology, College of Life Science, Hainan Normal UniversityHaikou, China; ^4^Department of Plant Science, College of Life Science, Jiangsu Normal UniversityXuzhou, China; ^5^Department of Crop Science, College of Agricultural and Food Science, Zhejiang Agricultural and Forestry UniversityHangzhou, China

**Keywords:** *Kandelia candel*, Na^+^ flux, superoxide anion, hydrogen peroxide, salt, catalase, superoxide dismutase

## Abstract

Na^+^ uptake and transport in *Kandelia candel* and antioxidative defense were investigated under rising NaCl stress from 100 to 300 mM. Salinized *K. candel* roots had a net Na^+^ efflux with a declined flux rate during an extended NaCl exposure. Na^+^ buildup in leaves enhanced H_2_O_2_ levels, superoxide dismutase (SOD) activity, and increased transcription of *CSD* gene encoding a Cu/Zn SOD. Sequence and subcellular localization analyses have revealed that KcCSD is a typical Cu/Zn SOD in chloroplast. The transgenic tobacco experimental system was used as a functional genetics model to test the effect of KcCSD on salinity tolerance. *KcCSD*-transgenic lines were more Na^+^ tolerant than wild-type (WT) tobacco in terms of lipid peroxidation, root growth, and survival rate. In the latter, 100 mM NaCl led to a remarkable reduction in chlorophyll content and a/b ratio, decreased maximal chlorophyll *a* fluorescence, and photochemical efficiency of photosystem II. NaCl stress in WT resulted from H_2_O_2_ burst in chloroplast. Na^+^ injury to chloroplast was less pronounced in *KcCSD*-transgenic plants due to upregulated antioxidant defense. *KcCSD*-transgenic tobacco enhanced SOD activity by an increment in SOD isoenzymes under 100 mM NaCl stress from 24 h to 7 day. Catalase activity rose in *KcCSD* overexpressing tobacco plants. *KcCSD*-transgenic plants better scavenged NaCl-elicited reactive oxygen species (ROS) compared to WT ones. In conclusion, *K. candel* effectively excluded Na^+^ in roots during a short exposure; and increased *CSD* expression to reduce ROS in chloroplast in a long-term and high saline environment.

## Introduction

NaCl-exposed plants accumulate a high level of Na^+^ in roots and leaves regardless of Na^+^-resistant or -sensitive species (Chen and Polle, 2010; Polle and Chen, 2014). Na^+^ excess would lead to ionic imbalance, causing Na^+^ injury (Volkov et al., 2004). To avoid excessive buildup of Na^+^, non-secretor mangrove species (*Kandelia candel*) can maintain a high capacity to restrict Na^+^ uptake and transport after NaCl exposure (Li et al., 2008). *K. candel* roots exhibited Na^+^ efflux by increasing H^+^ influx, indicating that Na^+^ efflux resulted from active Na^+^ exclusion across the plasma membrane (Lu et al., 2013; Lang et al., 2014). However, its roots and shoots could accumulate large amount of Na^+^ under a long-term of increasing salinity (Li et al., 2008). This implies that the capacity for Na^+^ exclusion decreased in salinized roots. However, this hypothesis needs further investigations.

In addition to ion-specific toxicity, Na^+^ accumulation in leaves leads to oxidative stress by the production of reactive oxygen species (ROS) in trees (Wang et al., 2007, 2008). Superoxide anions (O^−^_2_) are generated as a byproduct of electron transport mainly in mitochondria or chloroplasts, which results in subsequent formation of hydrogen peroxide (H_2_O_2_) and hydroxyl radicals (OH^.−^) by a successive univalent reduction of oxygen (O_2_) via chemical and enzymatic reactions (Asada, 1999; Apel and Hirt, 2004). Excessive ROS are potentially harmful to plant cells because of inactivating photosystem (PS) I and PS II (Jakob and Heber, 1996), and causing oxidative damage to proteins, lipids, and nucleic acids (Apel and Hirt, 2004). *K. candel* plants have an oxygen scavenging system against ROS under NaCl stress. Proteomic analysis of its leaves revealed that superoxide dismutase (SOD) abundance increased in response to high NaCl at 450–600 mM (Wang et al., 2014). SODs constitute the first line of cellular defense against ROS by rapidly converting O^−^_2_ and water to H_2_O_2_ and O_2_ (Bowler et al., 1992; Fridovich, 1995). Furthermore, SOD contributes to minimizing OH^.−^ formed by Haber–Weiss or Fenton reactions (Bowler et al., 1992; Gutteridge and Halliwell, 2010). Wang et al. (2013) found that abiotic-stress proteins were up-regulated by NaCl in *K. candel* chloroplasts. However, the protection of chloroplast Cu/Zn SOD (CSD) to salt tolerance is still poorly understood.

The objectives of this study were to investigate Na^+^ uptake and transport in *K. candel* plants and antioxidative defense under increasing NaCl salinity. Alterations of Na^+^ flux were recorded in young roots using a non-invasive ion flux technique. Transcriptional response of *CSD* to salinity was examined in *K. candel* leaves. Microarray analysis has shown that NaCl stress increases *KcCSD* expression (Hou, 2010). To clarify the role of *KcCSD* in salinity tolerance, *KcCSD* gene of chloroplast from *K. candel* was cloned and transferred to *Nicotiana tabacum*, a model system for investigating novel genes in salt tolerance (Han et al., 2013; Shen et al., 2013). In wild-type (WT) tobacco and *KcCSD*-overexpressing lines, ROS accumulation in leaf cells and activities of SOD, catalase (CAT), and ascorbate peroxidase (APX) were examined under 100 mM NaCl stress. This study could provide scientific evidence of KcCSD protection for antioxidant defense in chloroplast.

## Materials and methods

### Plant material and treatments

Uniform mature hypocotyls of *K. candel* were obtained from Dongzhai Harbor in Hainan Province of China (19°51′N, 110°24′E). Uniform hypocotyls from the same tree were planted in 5-L pots containing sand. Potted plants were placed in a greenhouse at Beijing Forestry University and fertilized with half-strength Hoagland's nutrient solution (Hoagland and Arnon, 1950) every 2 weeks at 20–25°C under a 12-h daily photoperiod with 200–300 μmol m^−2^s^−1^. Plants were 25–30 cm high and had four pair leaves after 3 months of culture (Supplementary Figure S1). These plants were raised in Hoagland's nutrient solution without the addition of NaCl. Mangrove plants were in good physiological state since shoots and roots retained abundant salts (Li et al., 2008; Lu et al., 2013). NaCl treatment was started from 100 mM and increased stepwise by 100 mM weekly, until reaching 300 mM, which could avoid osmotic shock effects of NaCl saline on the plants (Li et al., 2008). Na^+^ flux in roots was recorded weekly and ion concentrations in roots and shoots were measured at low (100 mM) and high saline (300 mM) treatments, respectively. H_2_O_2_, *CSD* expression and SOD activity in leaves were measured after 8 h, 24 h, and 1, 2, and 3 weeks of NaCl treatment. The second pair leaves were harvested, quickly frozen in liquid nitrogen, and used for quantitative real-time PCR assays, SOD activity, and H_2_O_2_ measurements.

### Na^+^ flux recording in *K. candel* roots

Steady-state fluxes of Na^+^ were measured using non-invasive micro-test technique (NMT-YG-100, Younger USA LLC, Amherst, MA, USA). Na^+^ microelectrodes were prepared and calibrated as previously described (Lu et al., 2013; Lang et al., 2014). Length of primary roots of *K. candel* seedlings ranged from 1 to 5 cm (Supplementary Figure S1). Young roots with apices of 2–3 cm were excised from control and salinized plants for Na^+^ flux determination. Steady flux profiles of Na^+^ were measured along the root axis at the apical zones, where a vigorous flux was usually observed in woody plants (Sun et al., 2009a,b; Lu et al., 2013; Lang et al., 2014). Roots were rinsed with redistilled water and immediately incubated in measuring solution (with 0.1 mM Na^+^) for 30 min equilibration. The basic Na^+^ measuring solution (with low interfering ions of Ca^2+^ and K^+^, Cuin et al., 2011) was 0.1 mM NaCl, MgCl_2_, and CaCl_2_, and 0.5 mM KCl at pH 6.0. Roots were immobilized on the bottom of the chamber. Then ion flux recordings started 200 μm from the apex and conducted along root axis until 2000 μm with an interval of 300 or 500 μm. Ionic flux rates were obtained using MageFlux developed by Yue Xu (1995) (http://www.youngerusa.com).

### Ion analysis

Roots, leaves, stems, and hypocotyls were harvested from control and NaCl-stressed *K. candel* plants, and oven-dried to constant weight at 65°C for 4 days, ground and passed through a 1.0-mm sieve. Samples were digested by H_2_SO_4_-H_2_O_2_, and Na^+^ concentration was measured using an atomic absorption spectrophotometer (Perkin-Elmer 2280, PerkinElmer, Inc., Wellesley Hills, MA, USA) (Lu et al., 2013).

### RNA extraction and real-time PCR analysis

Total RNA was extracted from *K. candel* leaves (the second pair leaves) with a modified hot borate method (Wan and Wilkins, 1994). This protocol is commonly used for isolating RNA from plant tissues rich in polyphenols and polysaccharides. For tobacco, total RNA was extracted from the 3rd–4th mature leaves from the top using the Total RNA Extraction Kit (QBio Technologies Inc.). The integrity of total RNA was determined by running samples on 1.0% formaldehyde agarose gels stained with ethidium bromide. The quantity/yield of total RNA was estimated spectrophotometrically at 230, 260, 280 nm (NanoDrop 2000 spectrophotometer, Thermo Scientific, Wilmington, USA). The first strand was synthesized from 2 μg of total RNA using M-MLV reverse transcriptase (Promega, Madison, USA) and oligo (d_T_) 12–15 primer at 42°C for 1 h. The real-time PCR conditions were 10 min at 95°C, 35 cycles of 95°C for 30 s, 55°C for 30 s, 72°C for 30 s, and 10 min at 72°C. The primers used for *KcCSD* were 5′-ATTAGCAACTATGTTTCCCA-3′ (forward), and 5′-CTACAACGGTGAATGTTC-3′ (reverse). The real-time PCR data in *K. candel* were normalized against *Tubulin: Tubulin* -F, 5′-TGCCCAAGGATGTGAACG-3′; and *Tubulin*-R, 5′-CCATACCCTCACCCACAT-3′. In tobacco, *EF1α* was used as the internal control (forward, 5′-GCTGTGAGGGACATGCGTCAAA-3′; and reverse, 5′-GTAGTAGATCGCGAGTACCACCA-3′).

The real-time PCR analysis was performed in a Real-time PCR System (MJ Opticon2 Bio-Rad). The relative level of expression was quantified using MJ Opticon Monitor software (Bio-Rad, Hercules, CA, USA). The expression of the target genes were normalized to the expression level of the reference gene (*Tubulin* in *K. candel* and *EF1α* in tobacco) using the 2^−ΔΔC_T_^ method (Livak and Schmittgen, 2001).

### Sequence alignment and phylogenetic tree

Full-length amino acid sequences of Cu/Zn SOD were aligned using ClustalW2 online (http://www.ebi.ac.uk/Tools/msa/clustalw2/). Amino acid sequences of chloroplast transit peptide were predicted by ChloroP (http://www.cbs.dtu.dk/services/TargetP/; http://www.cbs.dtu.dk/services/ChloroP/) (Emanuelsson et al., 1999, 2007). Phylogenetic tree was generated using the Neighbor–Joining (NJ) method in MEGA version 6.0 software (bootstrap analysis with 1000 replicates). The accession numbers of Cu/Zn SOD protein sequences used in multiple sequence alignment and phylogenetic analysis are provided in Supplementary Table S1.

### Plasmid constructs

The open reading frame (ORF) of *KcCSD* (GenBank accession number KP143653) was amplified by PCR from *K. candel* cDNA using specific primers (forward, 5′-ATGCAAGCGGTAGTTGCG-3′; reverse, 5′-TAACACTGGTGTCAACCCAACAAC-3′). The PCR fragment was first cloned into the vector pMD18-T (Takara, Dalian, China) and verified by sequencing. The ORF of *KcCSD* was released by digestion with *Eco*RI and *Hin*dIII and introduced into the expression vector, pCAMBIA2300, under the control of the constitutive cauliflower mosaic virus 35S promoter.

### Overexpression of *KcCSD* in transgenic tobacco lines

To further analyze the functions of KcCSD upon NaCl stress, *KcCSD* was transferred to tobacco plants via *Agrobacterium*-mediated gene transfer. The pCAMBIA2300-*KcCSD* construct was transferred to a strain of *Agrobacterium tumefaciens* (LBA4404) with a freeze–thaw method. Tobacco was infected by the *Agrobacterium tumefaciens* using the leaf disc method (Horsch et al., 1985). The infected leaves were placed on MS medium (Murashige and Skoog, 1962) with no antibiotic for 2–3 days and transferred to MS with 50 mg/L kanamycin. Individual kanamycin-resistant shoots were selected and shooted on MS medium with no growth regulators or antibiotic. More than 10 independently transformed plants were selected for *KcCSD* expression assay. Those overexpressing *KcCSD* were used for further study (L7 and L8; Supplementary Figure S2). WT and *KcCSD*-overexpressed plants were kept in the greenhouse to yield seeds. Using PCR, T2 generation of L7 and L8 was checked for the presence of the *KcCSD* gene.

T2 generation seeds of wild-type (WT) and transgenic lines (L7 and L8) were germinated on MS medium for 7 days, then subjected to 0 or 150 mM NaCl for 7 days. Root length, survival rates, and H_2_O_2_ level in chloroplast were measured in WT and transgenic plants. The capacity to control ROS was compared between WT and transgene tobacco under NaCl stress. Four-weeks old rooted plants of WT and transgenic lines were transferred to 1/4 Hoagland's nutrient solution for 2-weeks acclimation, then exposed to 0 or 100 mM NaCl for 7 days. O^−^_2_ and H_2_O_2_ production, lipid peroxidation, and activity of antioxidant enzymes (SOD, CAT and APX), SOD isoenzymes were examined during NaCl treatment (100 mM, 7 day). Leaf photosynthesis, chlorophyll a fluorescence, chlorophyll content were measured in NaCl-stressed WT and transgenic plants.

### Subcellular localization of the GFP fusion proteins

To generate a translational fusion of KcCSD-GFP, the *KcCSD* was obtained by PCR using specific primers (forward 5′-ATGCAAGCGGTAGTTGCG-3′; reverse 5′-CACTGGTGTCAACCCAACAAC-3′). PCR products were cloned into the pMD18-T vector and sequenced. The resulting construct was digested by *Eco*RI and *Pst*I and introduced into the constructed pGreen0029-GFP vector driven by the 35S promoter. Vector-carrying 35S-driven GFP was used as free GFP control.

*KcCSD-GFP* was transiently transformed to mesophyll protoplasts of *Arabidopsis thaliana* due to a high frequency of gene transformation, as compared to the tobacco. Isolation of *Arabidopsis* mesophyll protoplasts and polyethylene glycol–mediated transformation were performed according to Yoo et al. (2007). Confocal images were obtained after 16–20 h of incubation. Fluorescence was examined with a Leica inverted fluorescence microscope (Leica Microsystems GmbH) at 510–535 nm for GFP and at 650–750 nm for chlorophyll.

### Malondialdehyde (MDA) content

Tobacco leaves (0.1 g, 3rd–5th mature leaves from the top) were homogenized in 1 ml of 0.1% trichloroacetic acid solution on ice. MDA content in WT and *KcCSD*-transgenic lines was determined by the thiobarbituric acid (TBA) reaction according to Heath and Packer (1968).

### Chlorophyll content, fluorescence parameters, and net photosynthetic rate

The 3rd–5th mature leaves from the top were used for chlorophyll contents, fluorescence, and photosynthesis measurements. Chlorophyll concentrations in WT and transgenic lines were measured according to Wellburn (1994) and Lichtenthaler (1987). Chlorophyll *a* fluorescence parameters were measured using a PAM fluorometer (Junior PAM, Walz, Germany). Plants were dark-adapted for 20 min to determine dark fluorescence yield (Fo), and then exposed to a single red pulse to determine maximal fluorescence yield (Fm) and Fv/Fm ratio using the formula (Fm-Fo)/Fm. Φ PSII, the PSII actual photochemical efficiency, was determined according to Wang et al. (2007). Net photosynthetic rate (Pn) was measured using a Li-6400 photosynthesis system (Li-Cor Inc., Lincoln, NE, USA) at 800 μmol photons m^−2^ s^−1^. Chamber air temperature was maintained at 25°C and CO_2_ concentration was 380 μL L^−1^.

### Total protein extraction and enzyme assays

Antioxidant enzymes were extracted from *K. candel* (3rd–4th mature leaves from the top) and tobacco leaves (3rd–5th mature leaves from the top) and measured according to Wang et al. (2007, 2008). The total SOD activity was determined by monitoring super-radical–induced reduction of nitro blue tetrazolium (NBT) at 560 nm (Giannopolitis and Ries, 1977; Wang et al., 2008; Shen et al., 2013). One unit of SOD (relative unit) was defined as the amount of enzyme that causes 50% inhibition of the reaction compared with a blank sample (Giannopolitis and Ries, 1977). CAT activity was determined spectrophotometrically by monitoring the disappearance of H_2_O_2_ at 240 nm for 1 min (Aebi, 1984). APX activity was determined by monitoring the H_2_O_2_-dependent oxidation of ascorbate at 290 nm for 1.5 min (Nakano and Asada, 1981).

### Native page of SOD isoenzymes

Salt-elicited alterations in SOD isoenzymes were examined after 7 days of NaCl treatment (100 mM). SOD was extracted from transgenic and WT tobacco leaves (3rd–5th mature leaves from the top) at 4°C (Beauchamp and Fridovich, 1971; Wang et al., 2007, 2008). Native PAGE of SOD was performed on a 7.5% separating gel and 3.9% stacking gel at 4°C. After electrophoresis, the SOD isoenzymes were differentiated on a pre-equilibrating gel for 30 min in 50 mM potassium phosphate buffer and 1 mM EDTA at pH 7.8. The gel was incubated in dark for 30 min in fresh staining solution of 50 mM potassium phosphate buffer (pH 7.8), 0.24 mM NBT, 33.2 μM riboflavin, and 0.2% tetramethylethylenediamine. Then it was illuminated with 400 μmol m^−2^ s^−1^ fluorescence until uniformly blue except areas with SOD activity.

### O^−^_2_ and H_2_O_2_ production in leaves

*In situ* accumulations of O^−^_2_ and H_2_O_2_ were examined with histochemical staining protocols. O^−^_2_ was detected with NBT (Dutilleul et al., 2003) and H_2_O_2_ with 3-3′-diaminobenzidine (DAB; Thordal-Christensen et al., 1997), respectively. For *in situ* staining of O^−^_2_, leaf discs (2 cm in diameter) were sampled from the second fully developed leaf on the top, and immediately vacuum infiltrated in 0.5 mg/ml NBT and 10 mM potassium phosphate buffer at pH 7.8. For the negative control, the NBT solution was supplemented with 10 U ml^−1^ SOD and 10 mM MnCl_2_ before the infiltration (Supplementary Figure S3). After being incubated in dark at room temperature for 1 h, samples were cleared in 90% ethanol at 70°C to remove chlorophyll. O^−^_2_ was visualized as a blue color at the site of NBT precipitation.

Leaf discs (2 cm in diameter) were vacuum infiltrated in 1 mg/ml DAB at pH 3.8, incubated in dark at room temperature for 14 h and transferred to 90% ethanol at 70°C until complete removal of chlorophyll and visualization of H_2_O_2_ as brown color at the site of DAB polymerization. Samples were stored and examined in 70% glycerol. For the negative control, 10 mM ascorbic acid was added into DAB solution for infiltration. Total leaf H_2_O_2_ was determined according to Wang et al. (2008).

### H_2_O_2_ detection in chloroplast

H_2_O_2_ in chloroplast was detected as described by Ramírez et al. (2013) with minor modifications. H_2_O_2_-specific fluorescent probe, H_2_DCF-DA, was an indicator of H_2_O_2_ (Sun et al., 2010a,b, 2012). WT and *KcCSD*-transgenic tobacco seedlings were germinated on MS for 7 days, then transferred to MS supplemented with 0 or 150 mM NaCl. Seedlings were vacuum infiltrated for 10 min in 10 μM H_2_DCF-DA (Sigma Aldrich) in 5 mM MES buffer at pH 5.7. After being incubated in dark for 20 min, leaf samples were washed with 5 mM MES for 30 min. Fluorescence was examined with a Leica inverted fluorescence microscope (Leica Microsystems GmbH) at 500–530 nm for H_2_DCF-DA and 650–750 nm for chlorophyll, respectively.

### Statistical analysis

All experimental data were subjected to SPSS (SPSS Statistics 17.0, 2008) for statistical tests and analyses. When *P* < 0.05, differences between means were considered significant unless otherwise stated.

## Results

### Na^+^ flux and Na^+^ concentrations in roots and shoots

**Figure 1:**
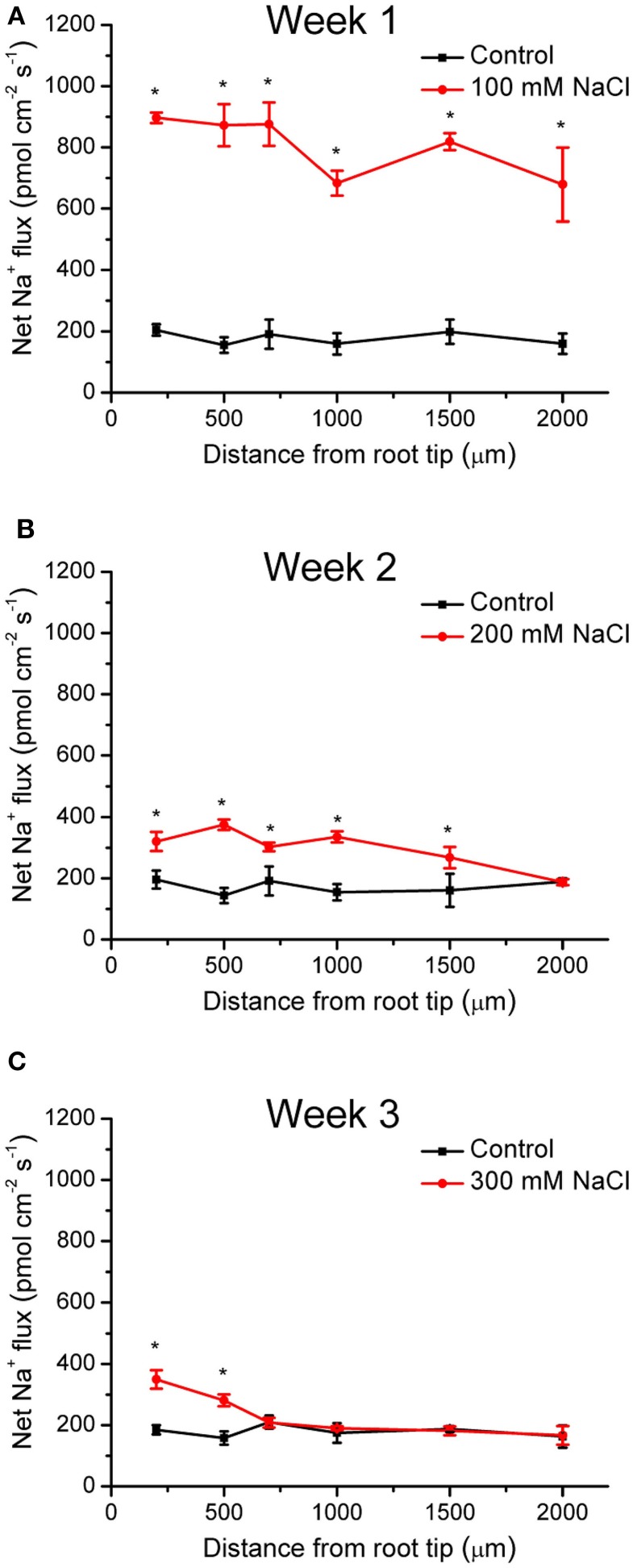
**Effects of NaCl on net Na^+^ fluxes in young roots of *K. candel***. Plants were treated by a rising NaCl concentration from 100 to 300 mM weekly for 3 weeks. The control was not exposed to NaCl. Steady Na^+^ flux profiles on week 1 **(A)**, week 2 **(B)**, and week 3 **(C)** were determined along root axis at apical zones, 200–2000 μm from the tip. Each point is the mean of 4–6 replicates. Bars represent standard error of the mean. ^*^P < 0.05, control and NaCl treatment.

**Figure 2:**
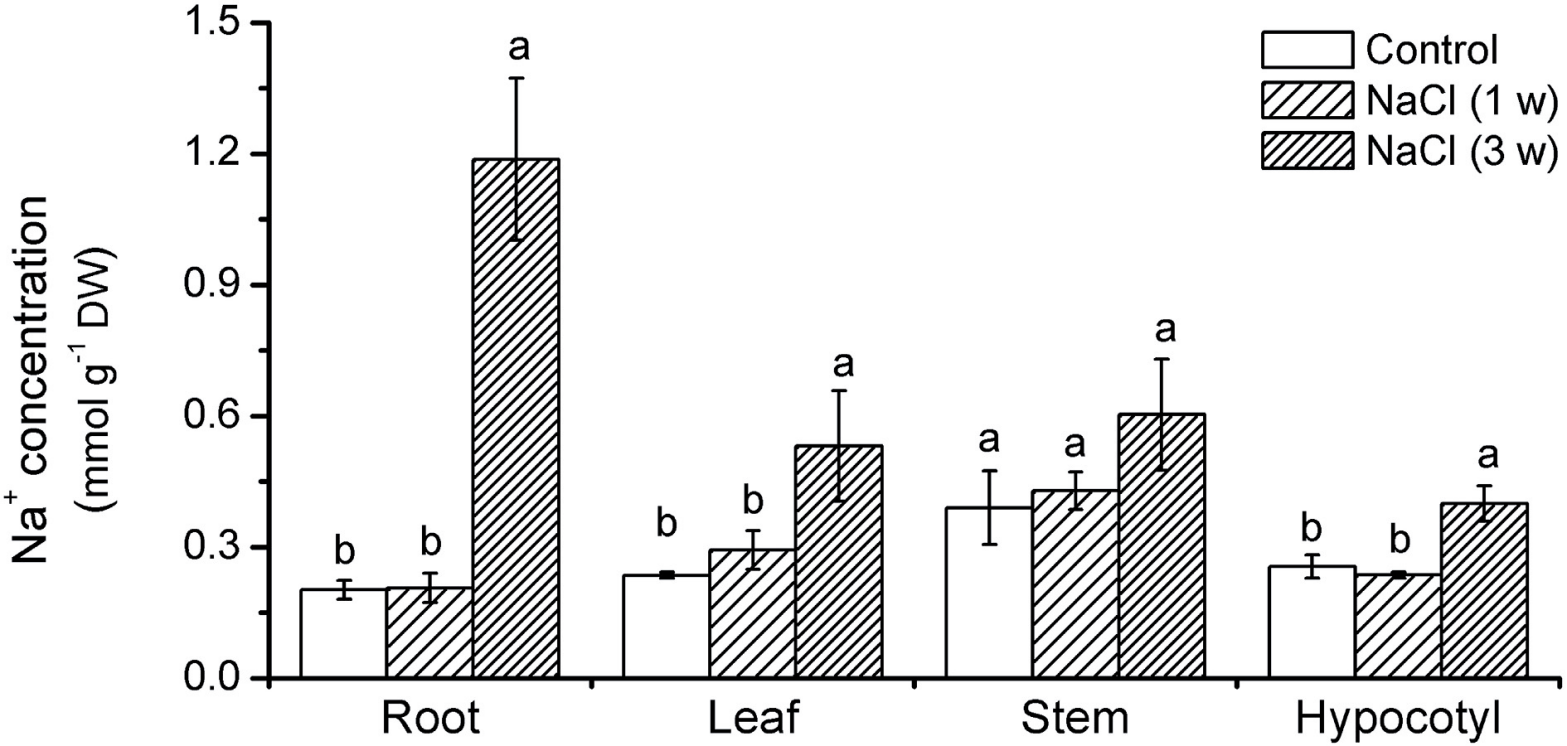
**Effects of NaCl on Na^+^ concentrations in roots and shoots in *K. candel***. Plants were treated by a rising NaCl concentration from 100 to 300 mM weekly for 3 weeks. Each column is the mean of 4–6 replicates. Bars represent standard error of the mean. Different letters denote significant differences at *P* < 0.05 between treatments in each organ.

During NaCl treatment, Na^+^ flux of *K. candel* was recorded along the root axes (200–2000 μm from the apex), in which a vigorous flux of Na^+^ was usually observed. Under control conditions, *K. candel* roots exhibited a stable and constant Na^+^ efflux with a mean flux of 176 pmol cm^−2^ s^−1^ (Figure 1). The Na^+^ efflux in control plants was due to Na^+^ previously accumulated by roots (Figure 2). However, Na^+^ efflux along roots was significantly increased by 4.5-fold in the first week of NaCl exposure (Figure 1). Flux rate in NaCl-stressed roots remarkably decreased with the extension of the exposure (Figure 1). The mean Na^+^ efflux in salinized roots was only 30% higher than the controls in the third week (Figure 1).

Na^+^ content in roots, hypocotyls, stems, and leaves significantly rose after 3 weeks treatment. It was 54–400% higher than that in the control (Figure 2). The Na^+^ accumulation was more pronounced in roots than in shoots (Figure 2).

### NaCl-elicited H_2_O_2_, SOD activity and expression of *KcCSD*

**Figure 3:**
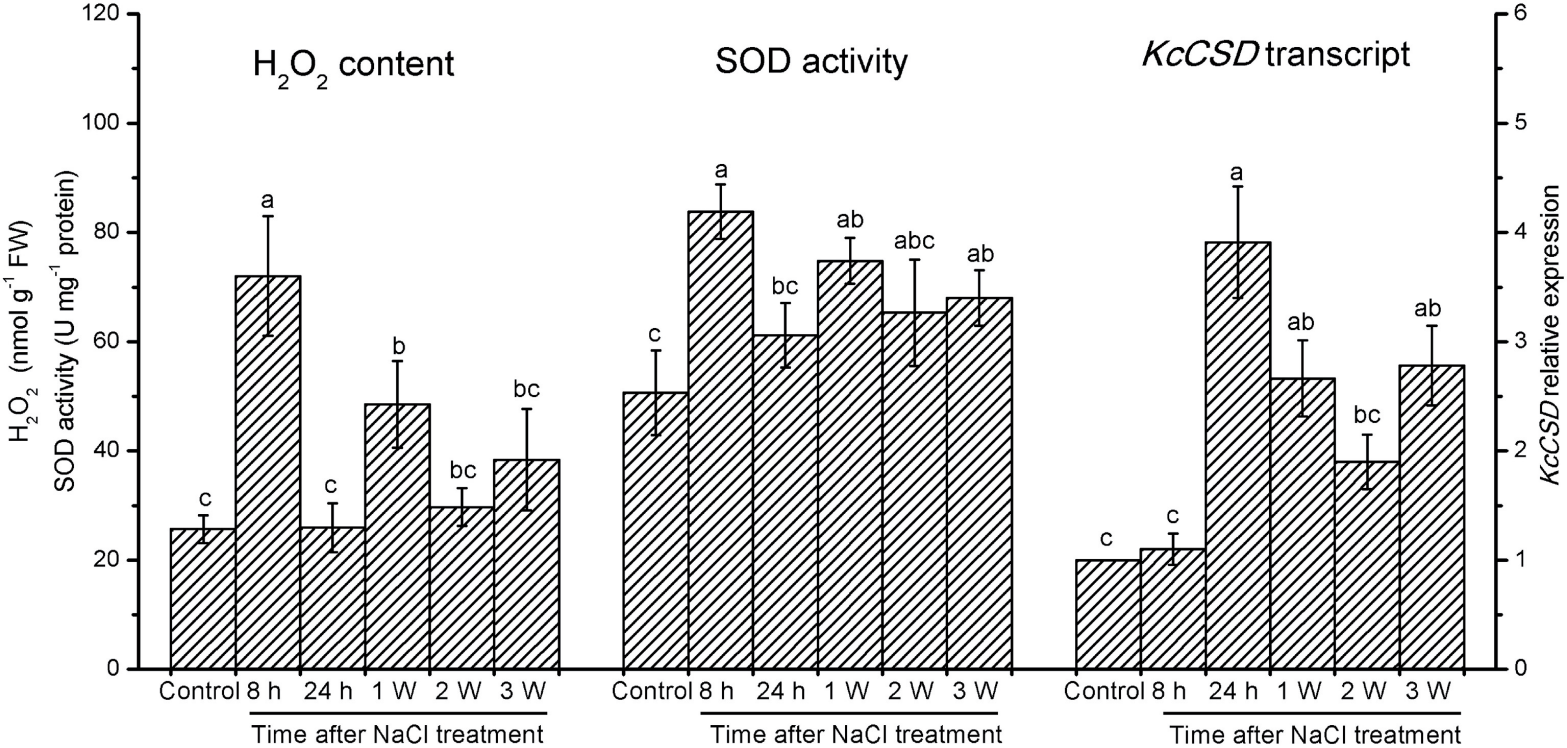
**Total concentration of H_2_O_2_, total SOD activity, and *KcCSD* expression in *K. candel* leaves under rising NaCl stress**. Plants were treated by a rising NaCl concentration from 100 to 300 mM weekly for 3 weeks. Each column is the mean of 4–6 replicates. Bars represent standard error of the mean. Different letters above columns represent significant differences at *P* < 0.05 during treatment.

NaCl salinity increased H_2_O_2_ levels in *K. candel* leaves although H_2_O_2_ levels fluctuated during 3 weeks experiment (Figure 3). Salinized *K. candel* enhanced SOD activity in its leaves (Figure 3). Quantitative real-time PCR (qRT-PCR) showed *KcCSD* upregulation during NaCl treatment (Figure 3). These results indicate a molecular and biochemical change in expression of *KcCSD* in salinized *K. candel*.

### *KcCSD* cloning and sequence analysis

**Figure 4:**
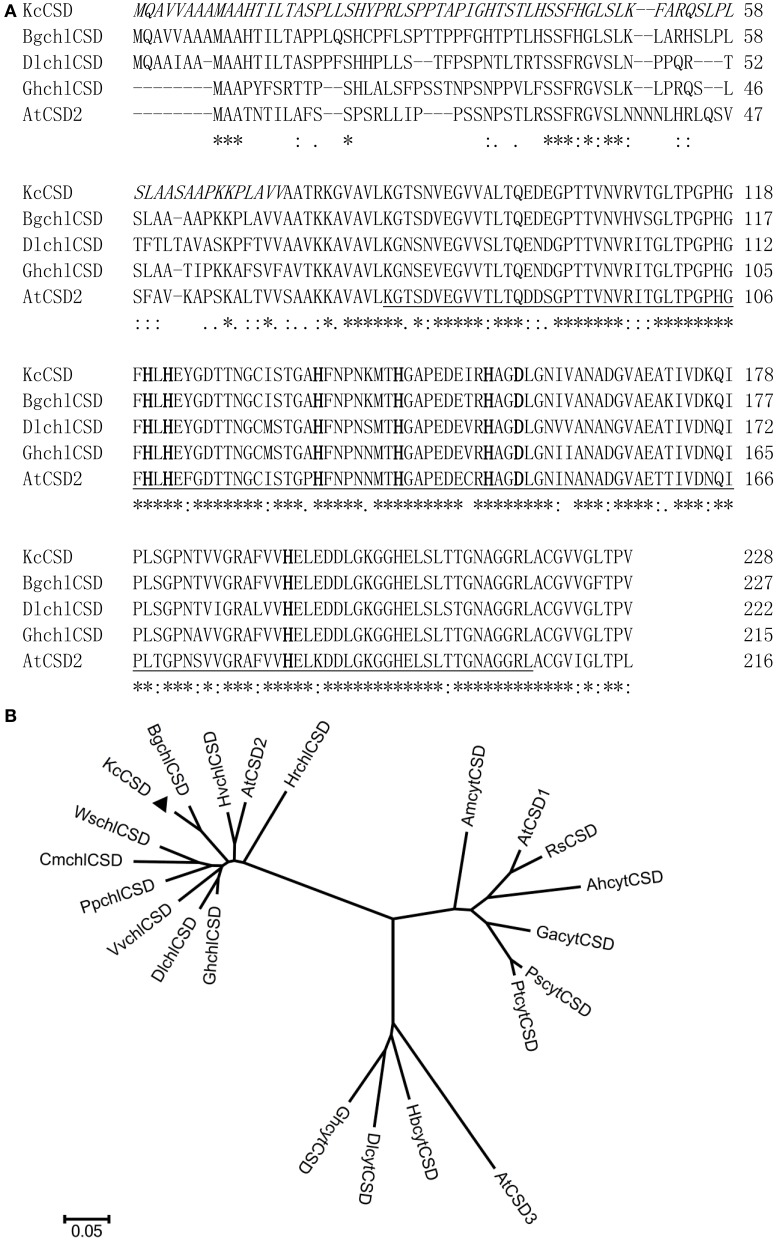
**Amino acid sequence and phylogenetic analysis of KcCSD. (A)** Amino acid sequences of Cu/Zn SOD from *Kandelia candle* (KcCSD), *Bruguiera gymnorrhiza* (BgchlCSD), *Dimocarpus longan* (DlchlCSD), *Gossypium hirsutum* chloroplast (GhchlCSD), and *Arabidopsis thaliana* chloroplast (AtCSD2). Asterisks (^*^) and dots (·, :) indicate identical and conserved amino acid residues, respectively. Italics are chloroplast transit peptide. Bolds indicate the conserved Cu^2+^ or Zn^2+^ binding site. Activity sites are underlined. **(B)** Neighbor-joined phylogenetic tree for CSD protein sequence (chloroplast CSDs with no chloroplast transit peptides) in various species. The alignment used for this analysis is available from the database (Supplementary Table S1). Different species acronyms are: *Ah*, *Amaranthus hypochondriacus*; *Am*, *Avicennia marina*; *At*, *Arabidopsis thaliana*; *Bg*, *Bruguiera gymnorrhiza*; *Br*, *Brassica rapa subsp. Pekinensis*; *Cm*, *Chenopodiastrum murale*; *Dl*, *Dimocarpus longan*; *Ga*, *Gossypium arboreum*; *Gh*, *Gossypium hirsutum*; *Hb*, *Hevea brasiliensis*; *Hr*, *Haberlea rhodopenis*; *Hv*, *Hordeum vulgare*; *Kc*, *Kandelia candel*; *Pp, Prunus persica*; *Ps*, *Populus suaveolens*; *Pt*, *Populus tremuloides*; *Rs*, *Raphanus sativus*; *Vv*, *Vitis vinifera*.

The cDNA sequence of *KcCSD* contains 687 bp with a predicted open reading frame (ORF) of 228 amino acids (Figure 4A). KcCSD protein conserves Cu^2+^ or Zn^2+^ binding site and active site (amino acids from 84 to 219), which catalyzes the conversion of O^−^_2_ to O_2_ (Figure 4A). It also contains a chloroplast transit peptide with a potential cleavage site at amino acid position 73 (Figure 4A). Comparative phylogenetic analysis of KcCSD has revealed that KcCSD is homologous to *Arabidopsis* CSD2 and other chloroplast CSDs from different species (Figure 4B). Collectively, KcCSD can be classified as CSD2, a chloroplast-targeted protein.

### Subcellular location of KcCSD

**Figure 5:**
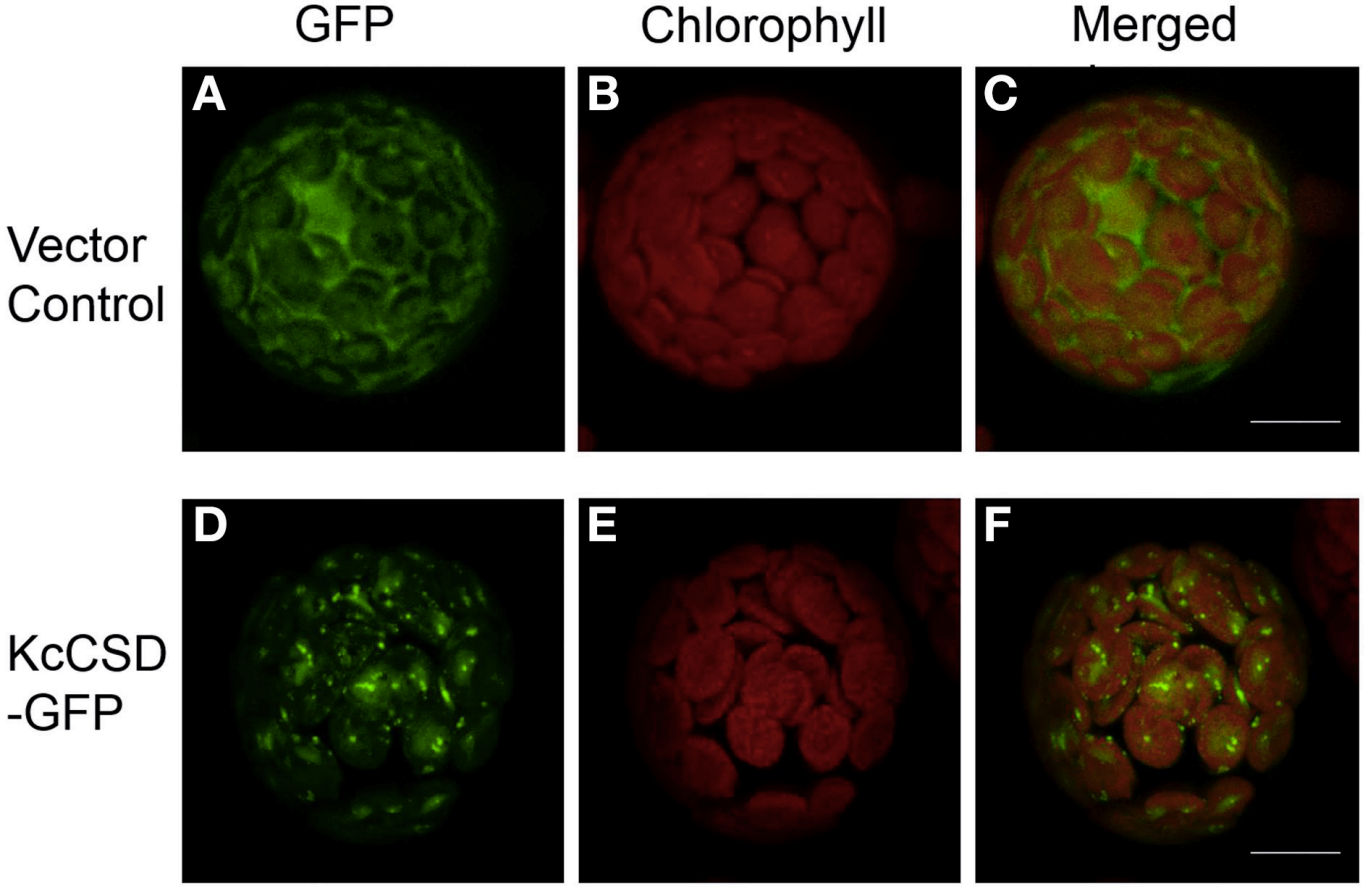
**Chloroplast subcellular localization of KcCSD**. Determined by transient expression of GFP alone (vector control; **A–C**) and a fusion KcCSD-GFP protein (KcCSD-GFP; **D–F**) in *Arabidopsis* protoplasts. Green fluorescence of GFP **(A,D)** and red auto-fluorescence of chlorophyll **(B,E)** were monitored separately using a confocal laser scanning microscope, and the two color fluorescence images **(C,F)** were merged. Bars = 10 μm.

A C-terminal translational construct was generated by the fusion of *KcCSD* to the green fluorescent protein (GFP) reporter gene. The construct was transiently expressed in *Arabidopsis* protoplasts (Figure 5). Fluorescence emitted by the GFP fusion of KcCSD overlapped chlorophyll autofluorescence, revealing that KcCSD was targeted to the chloroplast (Figures 5D–F). Fluorescence of the free GFP under the control of 35S promoter was distributed in cytoplasm of protoplasts, not merging with red autofluorescence from chloroplast (Figures 5A–C).

### Salinity tolerance of *KcCSD*-transgenic tobacco plants

Analysis by qRT-PCR identified a strong overexpression of *KcCSD* in the transgenic lines L1, L7, L8, and L10, but *KcCSD* was not detectable in the WT plants (Supplementary Figure S2). L7 and L8 transgenic lines showed a remarkably higher transcript abundance, indicating that *KcCSD* driven by the 35S promoter was more efficiently expressed in these lines, as compared to other ones. To testify the importance of *KcCSD* in enhancing NaCl tolerance, L7 and L8 were used for further NaCl treatment studies.

**Figure 6:**
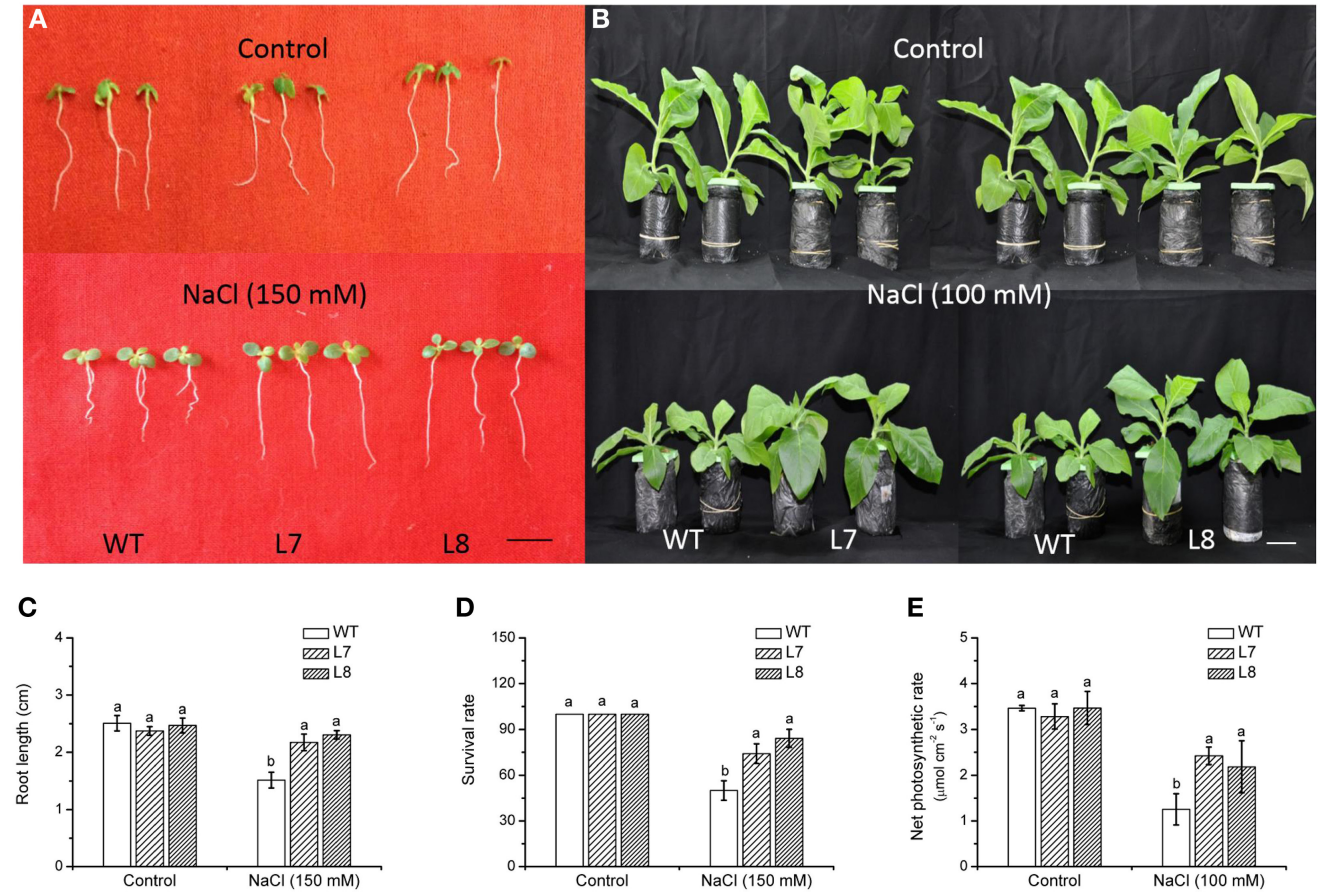
**NaCl tolerance of wild-type tobacco and *KcCSD*-transgenic lines**. Seeds of wild-type (WT) and transgenic lines (L7 and L8) were germinated on MS medium for 7 days, then supplemented with 0 or 150 mM NaCl for another 7 days. **(A,C,D)** Show root growth and survival rate of tobacco seedlings. **(B,E)** Show plant performance and net photosynthetic rate of tobacco plants after exposure to 0 or 100 mM NaCl for 7 **(E)** or 14 **(B)** days. Prior to NaCl treatment, 4-weeks old rooted plants of WT and transgenic lines were transferred to 1/4 Hoagland's nutrient solution for 2-weeks acclimation. In **(C**–**E)**, each column is the mean of 4–6 replicates. Bars represent standard error of the mean. Different letters above columns represent significant differences at *P* < 0.05 between WT and transgenic lines in control and NaCl treatments. Scale bars: A: 0.5 cm, B: 5 cm.

NaCl treatment (150 mM, 7 day) inhibited root growth and survival rate, but a more pronounced reduction occurred in WT (Figures 6A,C,D). Under hydroponic culture, WT plants showed a significant growth retardation compared to transgenic lines during a prolonged treatment (14 days; Figure 6B). Net photosynthetic rate (Pn) was decreased in WT and transgenic plants by salinity (Figure 6E). However, Pn was 74–93% higher in transgenic lines than in WT plants (Figure 6E). Under non-NaCl stress, both root and shoot growth of transgenic plants did not significantly differ from WT ones (Figure 6).

### Chlorophyll content and fluorescence

**Figure 7:**
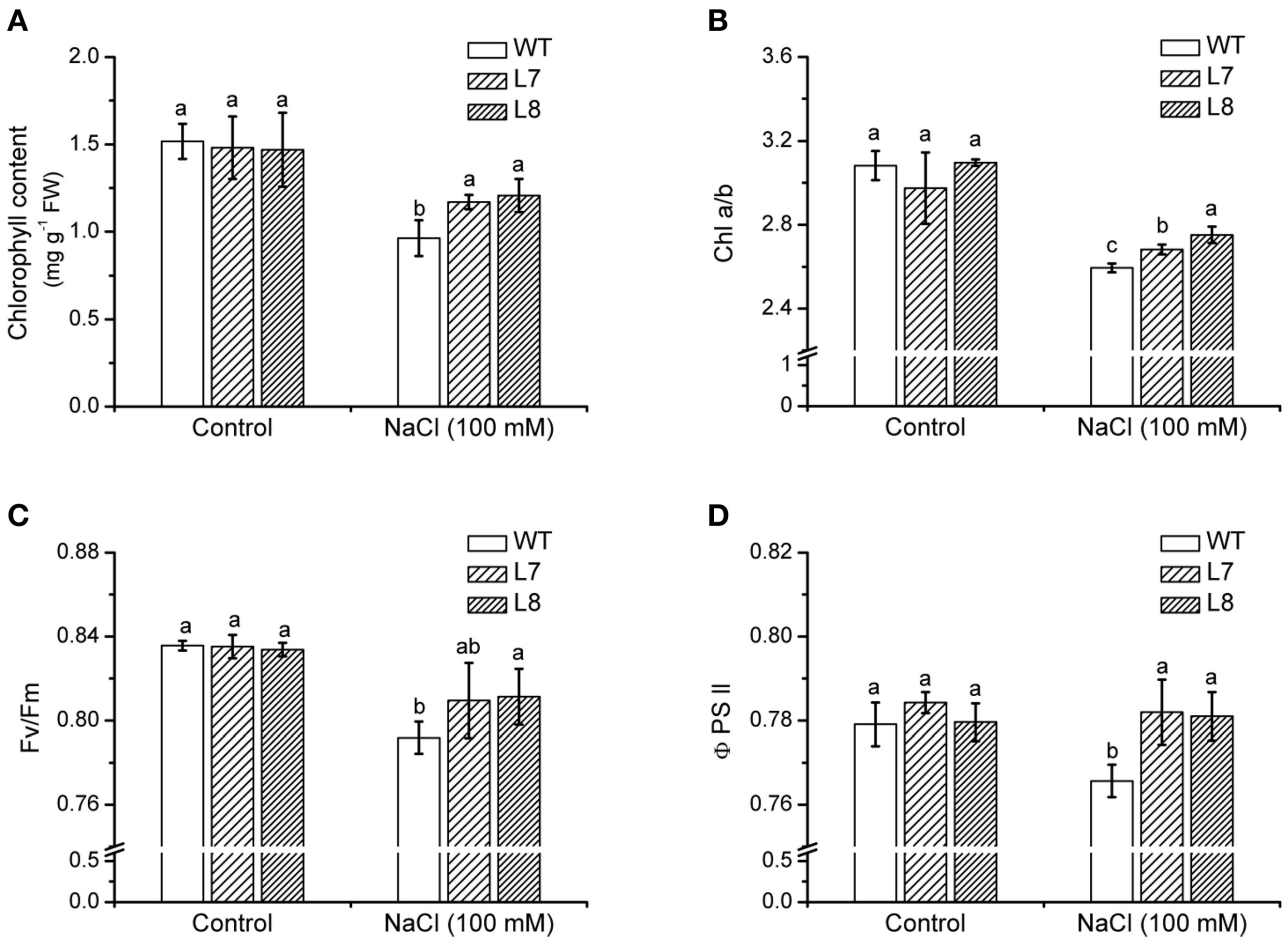
**Effects of NaCl on chlorophyll content and fluorescence in wild-type tobacco and *KcCSD*-transgenic lines**. Four-weeks old rooted plants of wild-type (WT) and transgenic lines (L7 and L8) were transferred to 1/4 Hoagland's nutrient solution for 2-weeks acclimation, then exposed to 0 or 100 mM NaCl for 7 days. **(A)** Chlorophyll content, **(B)** Chlorophyll a/b ratio, **(C)** Ratio of variable to maximal chlorophyll fluorescence (Fv/Fm), **(D)** Actual photochemical efficiency of PSII (Φ PSII). Each column is the mean of 4–6 replicates. Bars represent standard error of the mean. Different letters above columns represent significant differences at *P* < 0.05 between WT and transgenic lines in control and NaCl treatments.

NaCl treatment (100 mM) for 7 days resulted in a chlorophyll decline in tobacco plants (Figure 7A). However, it was more pronounced in WT than in transgenic lines L7 and L8 (Figure 7A). NaCl caused a decline in the chlorophyll a/b ratio by 14% in WT, which was greater than that in transgenic lines (Figure 7B).

After 100 mM NaCl treatment for a week, Fv/Fm was lowered in tobacco plants, but a more significant effect was observed in the WT ones (Figure 7C). Φ PSII, PSII actual photochemical efficiency (Maxwell and Johnson, 2000), in WT plants decreased remarkably by NaCl stress (Figure 7D). There was no similar change in *KcCSD*-transgenic lines (Figure 7D).

### O^−^_2_ and H_2_O_2_ production in tobacco leaves

**Figure 8:**
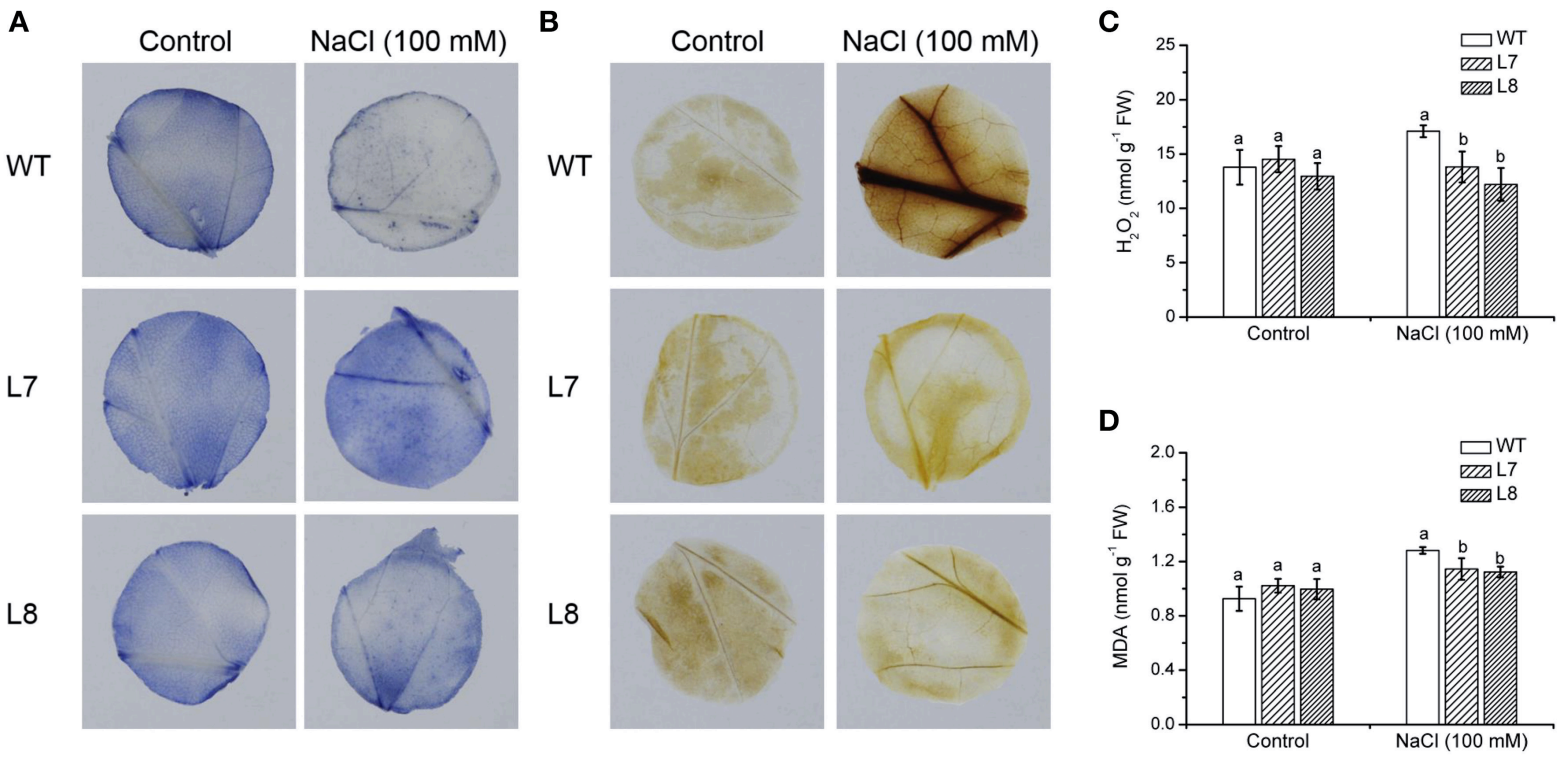
**Effects of NaCl on leaf ROS and malondialdehyde levels in wild-type tobacco and *KcCSD*-transgenic lines**. Four-weeks old rooted plants of wild-type (WT) and transgenic lines (L7 and L8) were transferred to 1/4 Hoagland's nutrient solution for 2-weeks acclimation, then exposed to 0 or 100 mM NaCl for 7 days. Leaf discs (2 cm in diameter) were sampled. **(A)** *In situ* O^−^_2_, **(B)** *In situ* H_2_O_2_, **(C)** Total H_2_O_2_, and **(D)** Malondialdehyde (MDA) content. In **(C**,**D)**, each column is the mean of 4–6 replicates. Bars represent standard error of the mean. Different letters above columns represent significant differences at *P* < 0.05 between WT and transgenic lines in control and NaCl treatments.

*In situ* O^−^_2_ production in leaves was detected by the reduction of nitro blue tetrazolium (NBT). Formazan deposits were visualized in leaf discs of WT and transgenic lines under no-NaCl conditions (Figure 8A). After 1 week exposure to 100 mM NaCl, more formazan precipitates appeared in tobacco leaves, especially in transgenic plants (Figure 8A). However, formazan formation in WT and *KcCSD*-transgenic plants was suppressed by SOD, the scavenger of O^−^_2_, irrespective of NaCl and control treatments (Supplementary Figure S3). This indicates that NBT was reduced to formazan specifically by the superoxide anions in WT and *KcCSD*-transgenic plants.

By means of DAB staining, H_2_O_2_ levels were visible in WT and *KcCSD*-transgenic lines (Figure 8B). Compared to the control, the intensity of red–brown staining significantly increased in NaCl-treated plants, and a more pronounced effect was observed in WT plants (Figure 8B). However, red–brown staining was absent in ascorbic acid-pretreated leaves (data not shown), indicating that the brownish staining was due to the reaction of DAB with H_2_O_2_. Total leaf H_2_O_2_ analysis showed a trend similar to that of *in situ* detection. H_2_O_2_ content in WT plants was increased by 16% under NaCl treatment, significantly higher than that in transgenic lines (Figure 8C).

### MDA content in leaves

NaCl-elicited increase in MDA, a marker of lipid peroxidation, was observed in both WT tobacco and *KcCSD*-transgenic lines (Figure 8D). However, WT showed 34% increase in MDA compared to transgenic lines L7 and L8 (10 and 17%; Figure 8D). This indicates that NaCl caused a more pronounced oxidative damage in WT than in transgene plants.

### H_2_O_2_ level in chloroplasts

**Figure 9:**
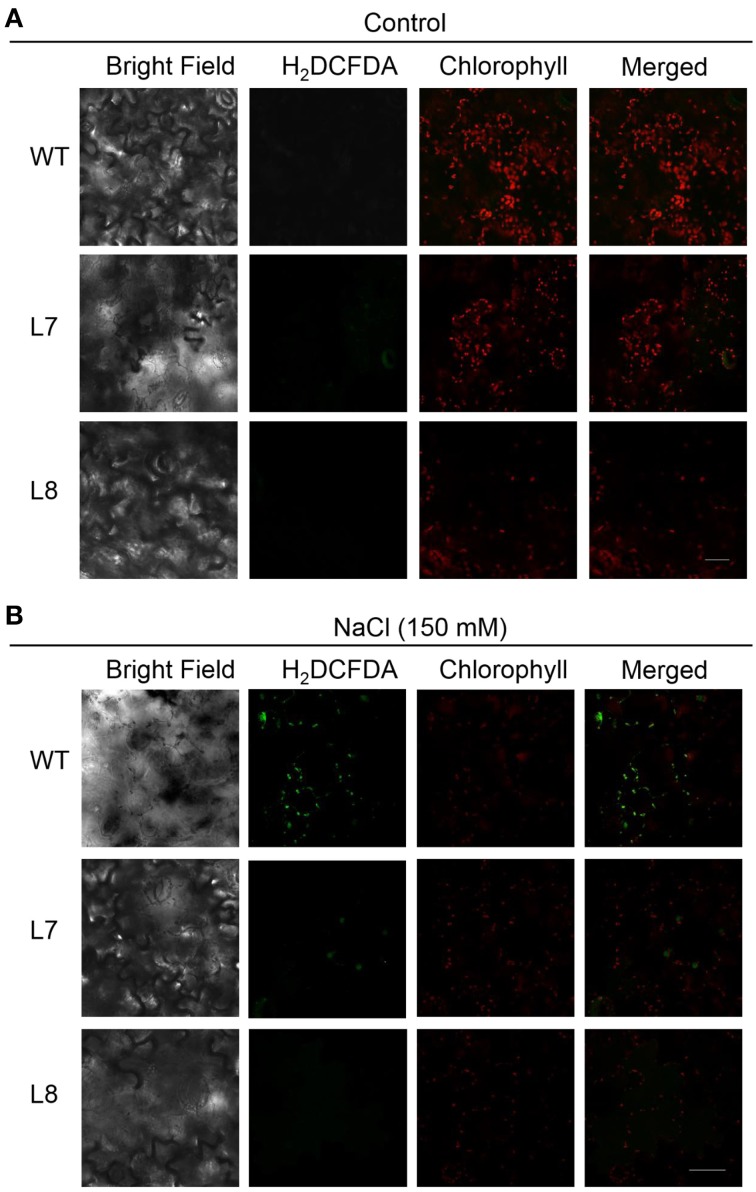
**Effect of NaCl on chloroplast H_2_O_2_ production in wild-type tobacco and *KcCSD*-transgenic lines**. Seeds of wild-type (WT) and transgenic lines (L7 and L8) were germinated on MS medium for 7 days, then supplemented with 0 **(A)** or 150 mM NaCl **(B)** for 7 days. Seedlings treated with or without NaCl were stained with 10 μM H_2_DCF-DA for H_2_O_2_ detection. Green fluorescence of H_2_DCF-DA and red auto-fluorescence of chlorophyll were monitored separately using a confocal laser scanning microscope, and two color fluorescence images were merged. Bars = 20 μm.

Confocal laser scanning microscopy analysis of leaf epidermal cells showed the same level of chlorophyll red auto-fluorescence in the WT tobacco and transgenic lines of the control (Figure 9A). In NaCl treatment, DCF-dependent green fluorescence occurred in NaCl-stressed tobacco plants (Figure 9B). WT plants had a higher fluorescent intensity than the transgenic lines (Figure 9B). Furthermore, the green fluorescence overlapped the red auto-fluorescence (Figure 9B), indicating that the NaCl-elicited H_2_O_2_ mainly originated from chloroplast. Excessive H_2_O_2_ accumulation in chloroplast would cause oxidative damage to WT leaves.

### Activity of antioxidant enzymes

**Figure 10:**
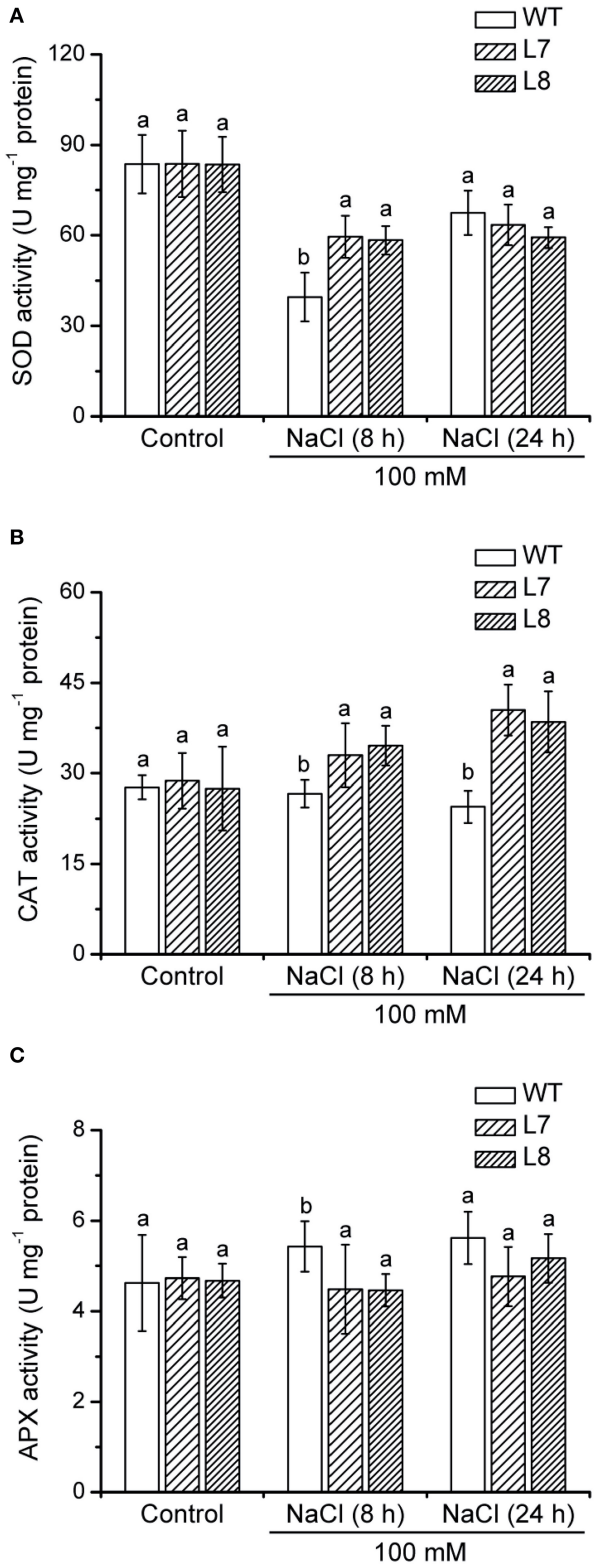
**Effect of NaCl on activities of antioxidant enzymes in wild-type tobacco and *KcCSD*-transgenic lines**. Four-weeks old rooted plants of wild-type (WT) and transgenic lines (L7 and L8) were transferred to 1/4 Hoagland's nutrient solution for 2-weeks acclimation. Hydroponically acclimated plants were subjected to 0 or 100 mM NaCl for 24 h. **(A)** Total SOD activity, **(B)** CAT activity, and **(C)** APX activity. Each column is the mean of 4–6 replicates. Bars represent standard error of the mean. Different letters above columns represent significant differences at *P* < 0.05 between wild-type and transgenic lines in control and NaCl treatments.

In control conditions, SOD, CAT, and APX activities were similar in WT tobacco and transgenic plants (Figure 10). CAT and SOD activities of *KcCSD*-transgenic lines L7 and L8 were 20 and 50% higher than in WT after 8 h of NaCl stress, respectively (Figures 10A,B). Moreover, transgenic plants displayed a significantly higher CAT activity than WT plants after 24 h stress (Figure 10B), indicating that the capacity to scavenge H_2_O_2_ was enhanced by NaCl treatment. NaCl did not significantly decrease APX activity in WT and transgenic lines during short salinity (Figure 10C).

**Figure 11:**
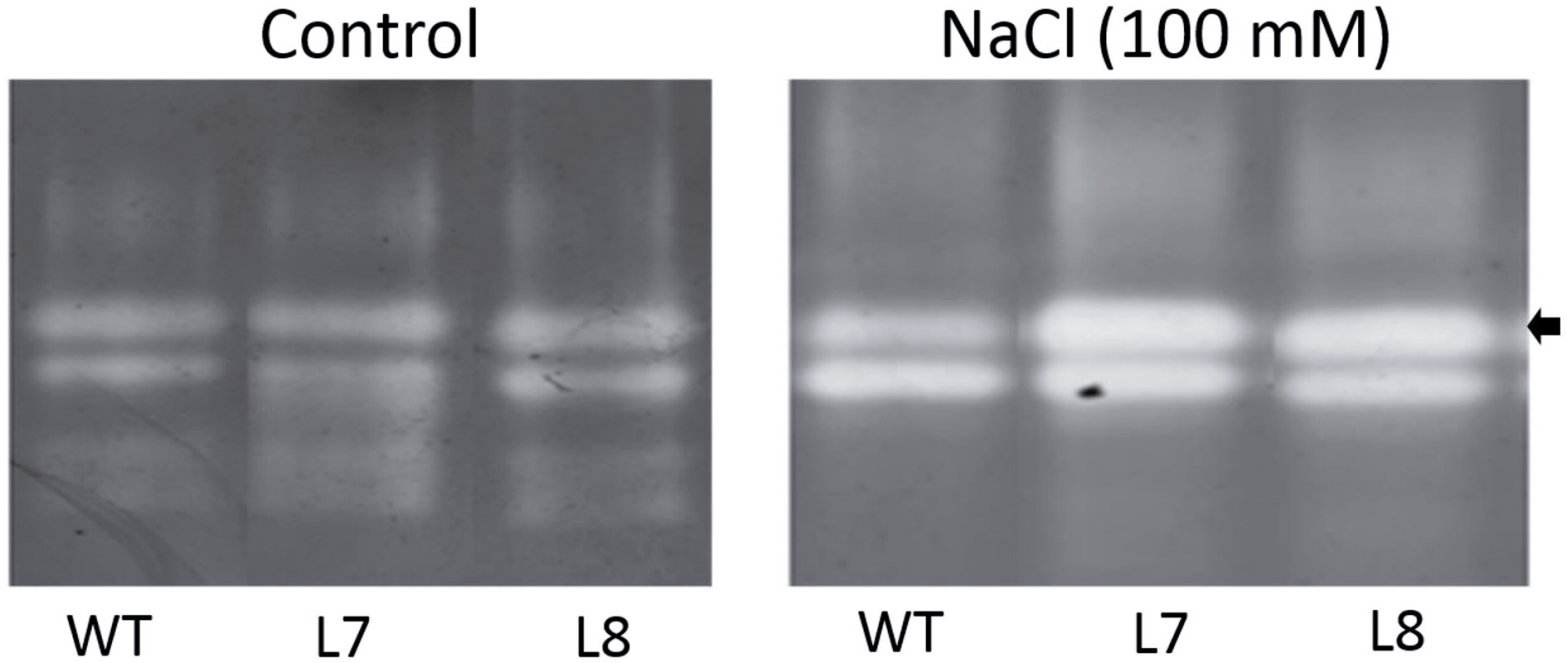
**Effect of NaCl on activities of SOD isoenzymes in leaves of wild-type tobacco and *KcCSD*-transgenic lines**. Four-weeks old rooted plants of wild-type (WT) and transgenic lines (L7 and L8) were transferred to 1/4 Hoagland's nutrient solution for 2-weeks acclimation. Hydroponically acclimated plants were subjected to 0 or 100 mM NaCl for 7 days. The arrow indicates remarkably increased SOD isoform in transgenic lines.

A 7-day 100 mM NaCl treatment increased activity of two dominant SOD isoenzymes in both transgenic lines and WT plants (Figure 11). Transgenic plants exhibited a higher increase in activity of one of four SOD isoenzymes than WT plants did under NaCl stress (arrowhead, Figure 11), indicating that overexpression of *KcCSD* in tobacco led to an increased antioxidant defense.

## Discussion

### Na^+^ accumulation and upregulation of *KcCSD* in *K. candel* leaves

Young roots of *K. candel* had a net Na^+^ efflux under rising NaCl stress from 100 to 300 mM (Figure 1), which agreed with previous results in mangrove (Lu et al., 2013; Lang et al., 2014). Root Na^+^ efflux resulted from active Na^+^/H^+^ antiport across the PM (Lu et al., 2013; Lang et al., 2014). Root Na^+^ exclusion was more pronounced in a short treatment (1 week) than in a long-term stress (2–3 weeks) (Figure 1). This indicates that Na^+^ extrusion capacity in *K. candel* roots declined with the exposure. As a result, large amount of Na^+^ accumulated in roots was transported to other organs (hypocotyl, stem, and leaf) (Figure 2). Excessive Na^+^ accumulation in *K. candel* leaves led to an increase of ROS (Figure 3). This was tallied with the findings in poplars (Wang et al., 2007, 2008). A limit in oxidative damage to photosynthetic apparatus and antioxidant enzyme activities could benefit for detoxifying Na^+^-elicited ROS in *Populus euphratica* (Wang et al., 2007, 2008). In the present work, salinized *K. candel* promoted *KcCSD* expression and subsequently enhanced SOD activity in leaves (Figure 3). Protein abundance of SOD in *K. candel* leaves might increase under a high level of NaCl (Wang et al., 2014). Thus, it could be inferred that *K. candel* would upregulate the antioxidant enzymes to deal with a long-term saline stress. To investigate the role of KcCSD in salinity tolerance, *KcCSD* gene was transferred to the model species *Nicotiana tabacum*. The transgenic tobacco overexpressing *KcCSD* resulted in a greater root length and survival rate than WT plants under NaCl stress (Figure 6). This finding is consistent with other studies on transgenic Chinese cabbage plants (Tseng et al., 2007). Rice plants overexpressing a cytosolic Cu/Zn SOD gene (*Avicennia marina*) also conferred salinity tolerance in transgene plants (Prashanth et al., 2008). In this study, *KcCSD* overexpression in tobacco reduced ROS in chloroplasts under NaCl stress. The results confirmed the protection of chloroplast Cu/Zn SOD from NaCl stress.

### *KcCSD* expression and ROS control in chloroplast

Phylogenetic tree and sequence analyses have verified that KcCSD is a chloroplast CSD (Figure 4). The deduced protein sequence has confirmed a high similarity to chloroplast CSDs from other species, such as *Bruguiera gymnorrhiza*, *Dimocarpus longan*, *Gossypium hirsutum*, and *Arabidopsis thaliana* (Figure 4). In agreement with sequence analysis, a subcellular location assay revealed that KcCSD protein is targeted to chloroplast (Figure 5). Similarly, *Arabidopsis* CSD2 localized in chloroplast (Kliebenstein et al., 1998; Huang et al., 2012). ROS analysis in transgene tobacco plants indicated that KcCSD was involved in protecting chloroplasts from Na^+^ damage. NaCl treatment caused a significant increase in leaf H_2_O_2_ (Figure 8) and WT tobacco chloroplast (Figure 9). The H_2_O_2_ burst in chloroplast resulted from the SOD-catalyzed conversion of O^−^_2_. It was formed predominantly at a high rate of electron transfer to O_2_ (Asada, 1999; Apel and Hirt, 2004). The Na^+^-induced oxidative damage occurred in WT tobacco leaves. It led to an increased MDA and declined Pn, chlorophyll content, chlorophyll a/b ratio, Fv/Fm, and Φ PSII (Figures 6–8). This was a result of high H_2_O_2_ in chloroplast (Figure 9; Stepien and Johnson, 2009). Excessive H_2_O_2_ has been shown to trigger membrane lipid peroxidation, and limit membrane lipid unsaturation and membrane protein polymerization (Bowler et al., 1992; Wang et al., 2007).

Compared to WT tobacco plants, *KcCSD*-transgenic plants accumulated less H_2_O_2_ in both leaves and chloroplast under NaCl stress (Figures 8, 9). Unexpectedly, NaCl-treated transgenic plants retained higher O^−^_2_ production than WT (Figure 8). The high O^−^_2_ in salinized transgenic plants was likely due to feedback activation of O^−^_2_ production system. A high conversion of O^−^_2_ to H_2_O_2_ in *KcCSD*-transgenic plants accelerated the electron transfer to O_2_ via photosynthetic electron transport chain, thus activating positive feedback production of O^−^_2_. A lower H_2_O_2_ in *KcCSD*-transgenic plants mainly resulted from an increased activity of antioxidant enzymes (Figures 10, 11). Under either 24-h or 7-day NaCl stress, *KcCSD*-transgenic tobacco plants increased total activity of SOD due to an increment of SOD isoenzymes (Figures 10, 11). Moreover, in transgenic plants, NaCl treatment rapidly increased CAT after 8 h of salinity (Figure 10). Increased activities of CAT arose from an increased production of O^−^_2_ and H_2_O_2_, as ROS are secondary messengers to induce antioxidant defenses (Desikan et al., 2001; Vranová et al., 2002). It is evidenced that the antioxidant enzymes SOD, CAT, and APX play a crucial role in maintaining O^−^_2_ and H_2_O_2_ balance in the plants (Payton et al., 2001). Hence, increased activity of SOD in transgenic plants promoted the conversion of O^−^_2_ to H_2_O_2_ (Bowler et al., 1992; Fridovich, 1995) and CAT activation. Consequently, increased CAT would assist transgenic plants in reducing NaCl-elicited H_2_O_2_ in leaf cells during an extended NaCl stress (Figures 8, 9). Similar findings were observed in *K. candel* under NaCl treatment. Li (2009) showed that *K. candel* increased activities of SOD, CAT, APX, and glutathione reductase (GR) in leaves to control ROS during NaCl stress.

## Conclusions

**Figure 12:**
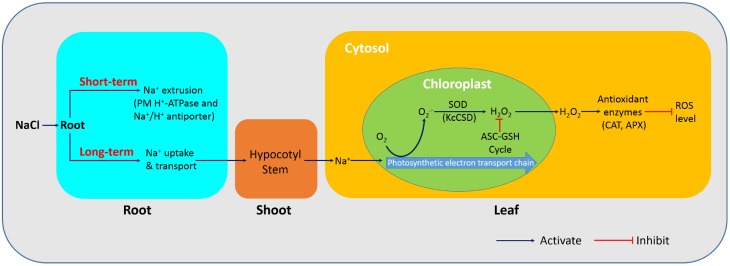
**Schematic model showing physiological mechanisms of *K. candel* adaptation to NaCl**. Under a short-term NaCl treatment, *K. candel* roots extrude Na^+^ by the plasma membrane Na^+^/H^+^ antiport system driven by H^+^-ATPase. During a prolonged stress, *K. candel* roots were unable to effectively limit Na^+^ uptake and transport, leading to Na^+^ accumulation in shoots. The buildup of Na^+^ in leaves favors the formation of O^−^_2_ via electron transport chain in chloroplasts. Salinized *K. candel* increases transcription of *CSD* gene encoding a copper/zinc superoxide dismutase (Cu/Zn SOD). An increased SOD activity in chloroplasts promotes the conversion of O^−^_2_ to H_2_O_2_; subsequently, chloroplasts scavenge and eliminate H_2_O_2_ via the ascorbate-glutathione (ASC-GSH) cycle (Asada, 1999). Moreover, the activated antioxidant enzymes, such as catalase (CAT), and ascorbate peroxidase (APX), readily scavenge H_2_O_2_ when the molecule diffuses across thylakoid membranes to the cytosol. As a result, the efficient scavenging of Na^+^-elicited ROS in chloroplasts avoids an occurrence of oxidative burst, and thus alleviates chloroplast injury from excessive ROS under the stress.

This study has revealed that *K. candel* has different physiological mechanisms to adapt to NaCl stress (Figure 12). As shown in the schematic model, *K. candel* roots could maintain a high capacity to extrude Na^+^ via a PM Na^+^/H^+^ antiport system driven by H^+^-ATPase. Under a prolonged stress, *K. candel* could activate its antioxidant system when roots were unable to effectively limit Na^+^ uptake and transport in the plant. The buildup of Na^+^ in leaves would favor the formation of O^−^_2_ via electron transport chain in chloroplast. *K. candel* could also upregulate *CSD* in leaves to detoxify Na^+^-elicited ROS and thus avoid an occurrence of oxidative burst. Ectopic expression of *KcCSD* in tobacco revealed that KcCSD could control ROS in chloroplast during NaCl stress. Accordingly, salinized *K. candel* increased Cu/Zn SOD activity to promote the conversion of O^−^_2_ to H_2_O_2_; subsequently, chloroplasts scavenged and eliminated H_2_O_2_ via the ascorbate-glutathione cycle (Asada, 1999). Moreover, the activated antioxidant enzymes, such as CAT and APX in the cytosol could readily scavenge the Na^+^-elicited H_2_O_2_ when H_2_O_2_ diffused across thylakoid membranes to the cytosol. The effective scavenge of Na^+^-elicited H_2_O_2_ in chloroplast alleviated chloroplast injury from excessive ROS under the stress. As a result, photochemical efficiency inhibition was physiologically mitigated and benefitted the plant for maintaining its photosynthesis and growth under the longer term salinity. Signaling network regulating *KcCSD* transcription under NaCl stress needs to be further investigated in the future.

## Author contributions

Xiaoshu Jing designed and performed the experiments, analyzed the experimental data, and prepared the manuscript. Peichen Hou, Yanjun Lu, Shurong Deng, Niya Li, Yang Wang, Yansha Han, and Tao Lang partly participated in the experiments (Peichen Hou: gene cloning and expression analysis of *KcCSD* in *Kandelia candel*, Yanjun Lu: Na^+^ fluxes recording in roots and Na^+^ concentrations, Shurong Deng subcellular location of KcCSD). Rui Zhao designed KcCSD-GFP construct. Rui Zhao, Jian Sun, Mingquan Ding, and Xin Shen conceived research plan. Shaoliang Chen designed research work and revised the manuscript. All authors have read and approved the final version of this manuscript.

### Conflict of interest statement

The authors declare that the research was conducted in the absence of any commercial or financial relationships that could be construed as a potential conflict of interest.
