# Supplementary figures and images for: Overexpression of copper/zinc superoxide dismutase from mangrove Kandelia candel in tobacco enhances salinity tolerance by the reduction of reactive oxygen species in chloroplast
Source: Front Plant Sci. 2015 Jan 22;6:23. doi: 10.3389/fpls.2015.00023 (PMC4302849; doi:10.3389/fpls.2015.00023)

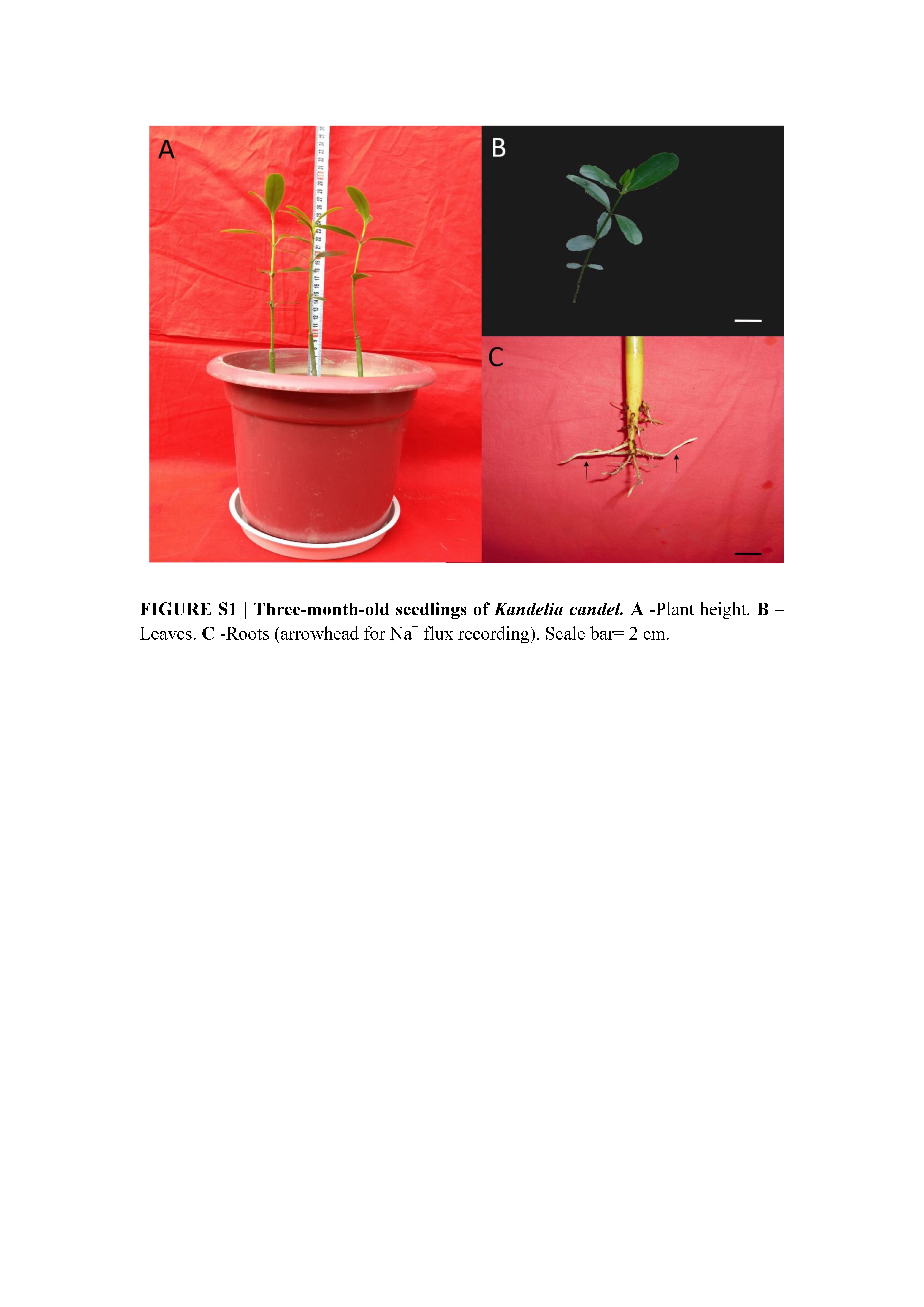

Supplement: Supplementary file 1 [file Image1.JPEG]

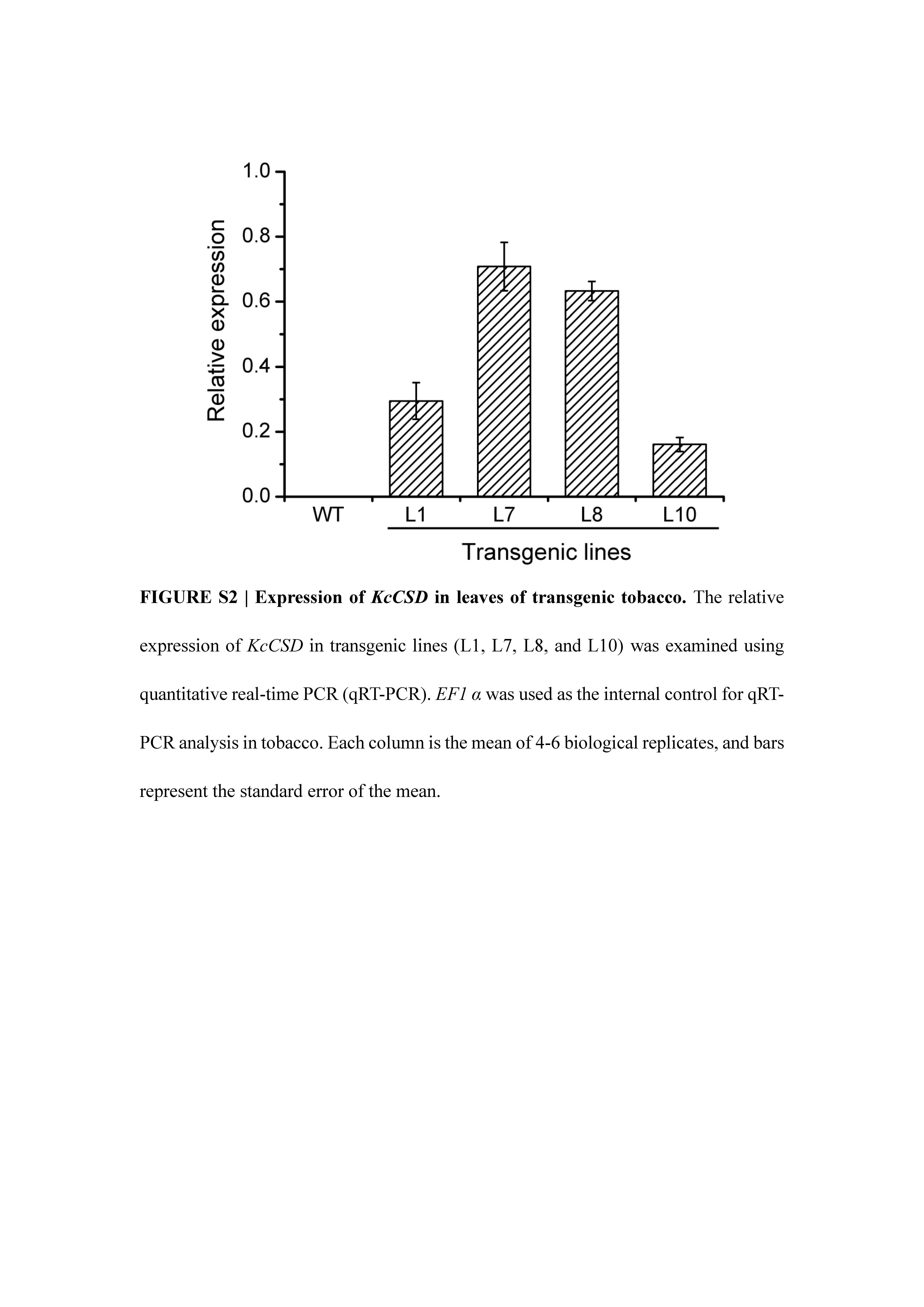

Supplement: Supplementary file 2 [file Image2.JPEG]

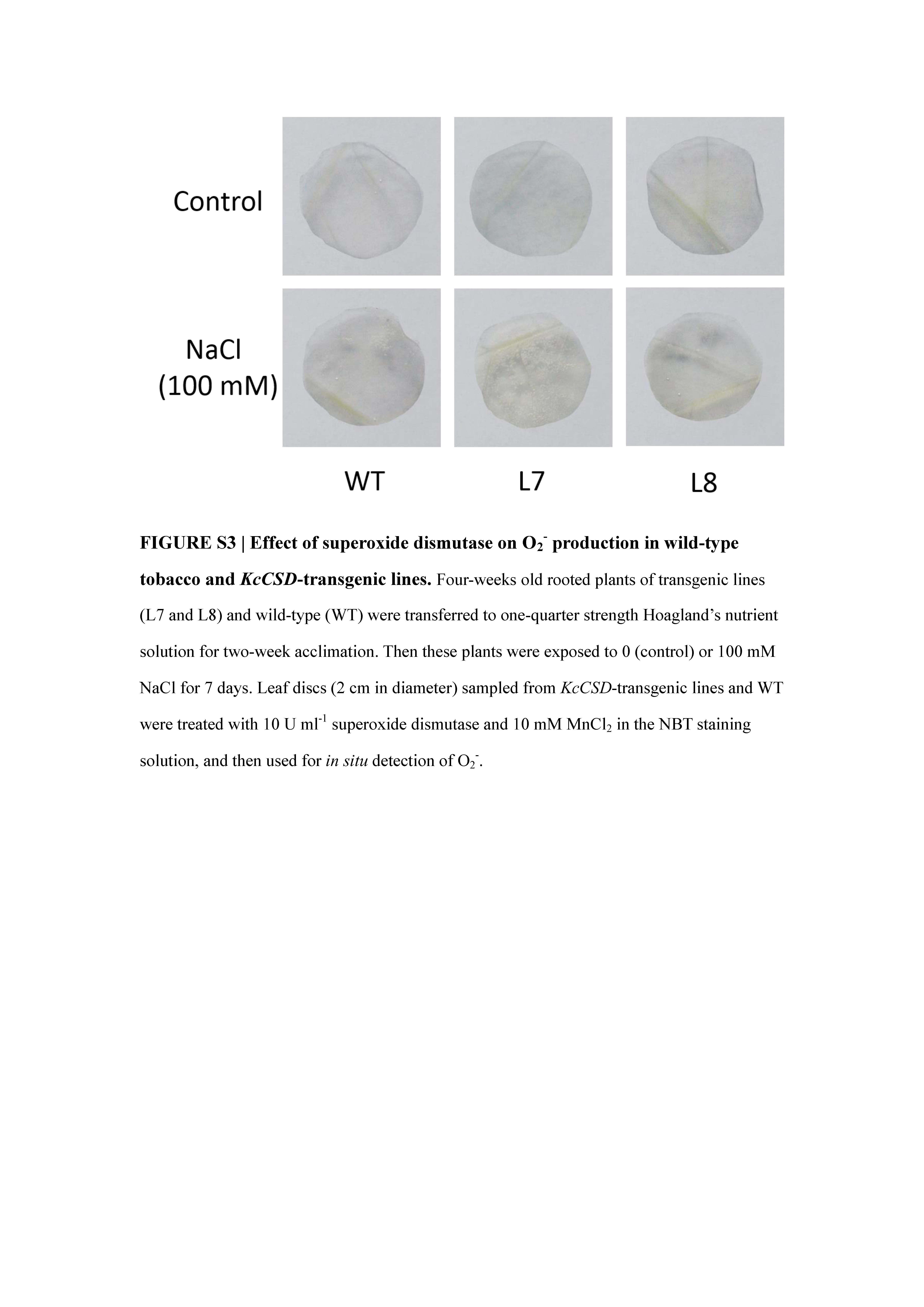

Supplement: Supplementary file 3 [file Image3.JPEG]
