# Supplementary material for: Overexpression of copper/zinc superoxide dismutase from mangrove Kandelia candel in tobacco enhances salinity tolerance by the reduction of reactive oxygen species in chloroplast
Source: Front Plant Sci. 2015 Jan 22;6:23. doi: 10.3389/fpls.2015.00023 (PMC4302849; doi:10.3389/fpls.2015.00023)
Supplement: Supplementary file 4 [file Table1.PDF]

TABLE S1| Accession numbers used in the amino acid sequence alignment and phylogenetic tree. The amino acid sequences of CSDs were obtained by a similarity search on the NCBI website (http://www.ncbi.nlm.nih.gov/).

Sequence name	Accession number	Sequence name	Accession number
AhcytCSD	ADH59419.1	GhchlCSD	ABL63518.1
AmcytCSD	AAK06837.1	GhcytCSD	ACC93640.1
AtCSD1	NP_001077494.1	HbcytCSD	ADR70869.1
AtCSD2	NP_565666.1	HrchlCSD	ADX36105.1
AtCSD3	NP_001119245.1	HvchlCSD	ADM47614.1
BgchlCSD	CAM98444.1	KcCSD	KP143653
BrcytCSD	AAC25568.1	PpchlCSD	AFH08801.1
CmchlCSD	ABO70347.1	PscytCSD	ABF48717.1
DlchlCSD	ADG26761.2	PtcytCSD	AAD01604.1
DlcytCSD	ADK70237.1	RsCSD	AAD05576.1
GacytCSD	ACI46676.1	VvchlCSD	NP_001268067.1
